# Dietary knowledge and perceptions in UK Pakistani/Bangladeshi communities: a survey on low-carbohydrate and intermittent fasting diets for diabetes prevention and management

**DOI:** 10.1017/S136898002610250X

**Published:** 2026-04-10

**Authors:** Grace Farhat, Musfirah Shariff, Charlotte Hopkinson, Zarish Yaqub, Sajda Majeed

**Affiliations:** 1 Faculty of Health and Education, Department of Health Professions, Manchester Metropolitan University, Manchester, UK; 2 Independent Patient and Public Engagement Contributor, Burnley, UK

**Keywords:** Type 2 diabetes, Low-carbohydrate diet, Intermittent fasting diet, South Asian population, Pakistani, Bangladeshi

## Abstract

**Objective:**

To assess knowledge and perceptions of low-carbohydrate and intermittent fasting diets among UK-based Pakistani and Bangladeshi individuals for type 2 diabetes prevention and management.

**Design:**

A cross-sectional survey.

**Setting:**

The survey was administered online using Jisc Online Surveys.

**Participants:**

Pakistani and Bangladeshi adults aged 18 and over who had lived in the UK for at least 1 year.

**Results:**

A total of 304 participants took part in the survey, of which 77 % (*n* 234) were females and 80·3 % (*n* 244) were Pakistanis. Intermittent fasting diets appeared to be somewhat more acceptable (*n* 107, 36 %) than low-carbohydrate diets (*n* 68, 22·8 %). Participants showed generally good dietary knowledge of carbohydrates and type 2 diabetes, although some gaps were identified. Key barriers to dietary change included reluctance to alter established eating habits as well as low motivation. Age, education and living arrangements were significant predictors of dietary knowledge and dietary preferences.

**Conclusions:**

These findings support carrying out future research to test culturally tailored interventions, with particular attention to intermittent fasting approaches. Multidisciplinary interventions that involve family members, offer flexible meal timing and present dietary guidance within familiar cultural contexts may improve acceptability and adherence and lead to long-term sustained benefits.

Type 2 diabetes (T2D) is highly prevalent among British South Asians, accounting for approximately 20 % of all T2D cases in the UK despite representing only about 7 % of the population^(1,2)^. This burden is particularly pronounced among British Pakistanis and Bangladeshis, who experience a sixfold higher risk of T2D, while Indian populations face a threefold increase^(3)^. Furthermore, British Pakistanis and Bangladeshis tend to develop faster progression to complications and have higher rates of non-communicable diseases such as CVD, contributing to increased morbidity and mortality^(4)^. This elevated risk of T2D is driven by a combination of genetic predisposition and socio-cultural and lifestyle factors, including traditional dietary patterns and physical inactivity^(5,6)^.

The Pakistani and Bangladeshi communities together comprise approximately 2 million individuals within the UK population and generally experience lower socio-economic status compared with other South Asian subgroups, as well as distinctly different dietary habits^(7,8)^. Despite this, the literature often fails to account for the heterogeneity within South Asian migrant populations. Significant subethnic differences in socio-economic conditions, cultural beliefs and dietary practices^(7–9)^ suggest that perceptions and understanding of dietary interventions are likely to vary, highlighting the need for tailored, evidence-based interventions for specific subgroups.

Low-calorie diets (∼850 kcal), in the form of total diet replacement, have transformed the management of T2D by promoting weight loss and facilitating diabetes remission^(10,11)^. While earlier feasibility studies and small-scale trials have demonstrated the acceptability of total diet replacement among South Asian populations^(12,13)^, there is an increasing emphasis on developing culturally tailored, food-based dietary interventions to prevent and manage T2D that enhance engagement and adherence^(10)^. Our previous patient and public engagement activity, which included South Asians of Pakistani and Bangladeshi origin, indicated a low acceptability of total diet replacement and a strong preference for culturally adapted, food-based approaches^(14)^.

Low-carbohydrate diet (LCD) and intermittent fasting (IF) have gained increased attention in recent years for their potential benefits in supporting weight loss and achieving remission of T2D. While various definitions of LCD exist, the Scientific Advisory Committee on Nutrition recommends a carbohydrate intake of ≤ 130 g/d to enhance glycaemic control and support weight management^(15)^, and its effectiveness has been documented in clinical studies^(16)^. Previous research indicates that the understanding of carbohydrates within South Asian communities is varied^(17)^, and although education plays a key role in enabling appropriate dietary carbohydrates, factors such as limited motivation and adherence to traditional cultural practices may act as barriers^(18)^. Perceptions of LCD have primarily been explored within white populations^(19)^, with limited research focusing on South Asian communities. As for IF, it encompasses a range of protocols, including time-restricted feeding, alternate-day fasting (e.g. the 5:2 diet) and periodic fasting, which have demonstrated promising effects on weight and glycaemic outcomes^(20,21)^. Given the Pakistani and Bangladeshi populations’ familiarity with religious fasting, it is anticipated that prior experience may help mitigate negative perceptions associated with IF^(22)^.

As health behaviours are dynamic and continually evolving^(18)^, gaining an up-to-date understanding of the acceptability and cultural alignment of dietary approaches within South Asian subgroups is essential to plan dietary interventions. This survey is therefore looking to assess knowledge, perceptions and acceptability of two emerging food-based diets for T2D prevention and management (LCD and IF) in a group of Pakistani and Bangladeshi participants living in the UK. The survey can help shape future studies using culturally tailored diets and, consequently, evidence-based recommendations to promote weight loss and glycaemic control in this population at high-risk of T2D.

## Methodology

### Study design

This study employed a cross-sectional survey design using an online platform (www.onlinesurveys.ac.uk). The survey included selected validated questions adapted from existing instruments^(19)^. First, a patient and public engagement activity was conducted to refine the survey. A panel of five English-speaking professionals (school teacher, nurse, community health worker, carer and business owner) from the Pakistani and Bangladeshi communities, as well as the public contributor, provided initial feedback. Concerns were raised regarding the length and complexity of the paper-based version, leading to the recommendation to use an online format exclusively to improve response rates. Some questions were removed, and additional questions were added to assess knowledge of diabetes. As per feedback, explanatory sections (‘More info’) were included for terms such as type 1 diabetes, T2D and IF.

Additionally, the panel expressed reservations about translating the survey into Urdu and Sylheti, citing potential loss of meaning. This view was supported by both a public contributor and a translator from the same ethnic background. To address potential language and digital literacy barriers and enable non-English speakers to take part, six bilingual (Urdu and English) public engagement volunteers were trained to support participants as needed.

Face and content validity were assessed by University staff and the public contributor. Readability was assessed by piloting the questionnaire in a small group of Pakistani and Bangladeshi individuals (*n* 5), who reported that it was easy to understand and could be completed within 20 min. The survey was then launched.

The estimated completion time ranged from 10 to 20 min, depending on participants’ prior knowledge and experience with dietary practices. Participants were allowed to return to the survey at any time to complete it. The survey was open from June 2022 to July 2025.

### Population

Individuals of Pakistani and Bangladeshi background residing in the UK for more than 1 year and aged 18 years or older were eligible to participate. Participants needed to have access to the internet and be able to read and write in English to complete the survey, which was available online and in English only. Exclusion criteria included individuals with cognitive barriers or those unwilling to provide consent. Recruitment was facilitated through social media platforms, places of worship, community gatherings and word of mouth. No identifiable personal information (e.g. name, date of birth) was collected.

### Data collection

Sociodemographic information (age group, gender, ethnicity, location, education level, profession and religion) was collected alongside data on participants’ medical history and dietary habits. The survey assessed participants’ understanding of carbohydrates, their knowledge of T2D, their current or past adherence to low-carbohydrate and IF diets and the motivations behind these dietary choices. Additionally, the study explored participants’ perceptions and preferences regarding each diet, focusing on their cultural, practical and behavioural acceptability and the perceived feasibility of adopting structured LCD and IF diets within the communities.

### Statistical analysis and sample size calculation

A minimum sample size of 259 participants was calculated based on an estimated adult Pakistani/Bangladeshi UK population of 1·5 million, a 2·5 % margin of error and a 99 % CI. To account for an anticipated 30 % rate of incomplete responses, the target recruitment was set at 337 participants.

Data were analysed using SPSS version 29.0. Descriptive statistics (frequencies and percentages) were used to summarise participant characteristics and key outcome variables. A multinomial logistic regression analysis was performed to examine how age, education level, profession, gender, living arrangements and UK birth status predict participants’ levels of knowledge and perceptions regarding LCD and IF diets. Significance was set at *P* ≤ 0·05.

## Results

A total of 304 individuals completed the survey, with 91·8 % completing it themselves and 8·2 % were assisted by public engagement volunteers. The majority identified as female (*n* 234, 77 %), of Pakistani background (80·3 %) and Muslim (91·8 %). Participants were predominantly aged between 18 and 34 years (55·2 %). A percentage of 21·4 % of respondents reported being unemployed or retired. Over half of the participants (58·7 %) were born outside the UK. Most participants reported living either with their parents (28·7 %) or with a partner, with or without children (44·9 %). In terms of educational attainment, the majority were either pursuing or had completed a degree (36·5 %) or a postgraduate qualification (34·2 %). A detailed summary of participant characteristics is presented in Table 1.

**Table 1.** Baseline characteristics of study participants

Characteristic	Total (*n* 304)		Pakistani (*n* 244)		Bangladeshi (*n* 45)		Mixed race* (*n* 15)	
	*n*	%	*n*	%	*n*	%	*n*	%
Age (years)								
18–24 years	84	27·6	63	25·8	14	31·1	7	46·7
25–34 years	84	27·6	72	29·5	10	22·2	4	26·7
35–44 years	62	20·4	47	19·3	12	26·7	1	6·7
45–54 years	36	11·8	28	11·5	7	15·6	1	6·7
55–64 years	16	5·3	14	5·7	0		2	13·3
65+ years	22	7·2	20	8·2	2	4·4	0	
Sex								
Male	68	22·4	51	20·9	10	22·2	7	46·7
Female	234	77	193	79·1	35	77·8	6	40
Non-binary	1	0·3	0		0		1	6·7
Prefer to self-describe	1	0·3	0		0		1	6·7
Ethnicity								
Pakistani	244	80·3						
Bangladeshi	45	14·8						
Mixed Pakistani/Bangladeshi background	3	1						
Mixed race (Pakistani/Bangladeshi and other ethnicity)	12	3·9						
Religion								
Muslim	279	91·8	224	91·8	43	95·6	12	80
Christian	18	5·9	17	7	1	2·2	0	
Hindu	2	0·7	0		1	2·2	1	6·7
No religion	5	1·6	3	1·2	0		1	6·7
Education level								
No qualifications	23	7·6	22	9	1	2·2	0	
GCSE/O-level	15	4·9	12	4·9	3	6·7	1	6·7
A-level or equivalent	43	14·1	31	12·7	11	24·4	1	6·7
Degree level	111	36·5	87	35·7	17	37·8	6	46·7
Postgraduate degree	104	34·2	86	35·2	12	26·7	6	40
Other	8	2·6	6	2·5	1	2·2	1	6·7
Employment status								
Unemployed/no income	65	21·4	59	24·2	6	13·3	0	
Health-related professions	41	13·5	32	13·1	4	8·9	5	33·3
Managers, directors and senior officials	32	10·5	26	10·7	5	11·1	1	6·7
Professional occupations (other than health-related)	53	17·4	39	16	10	22·2	4	26·7
Associate professional and technical occupations	13	4·3	11	4·5	2	4·4	0	
Administrative and secretarial occupations	9	3	6	2·5	1	2·2	2	13·3
Skilled trade occupations	11	3·6	10	4·1	1	2·2	0	
Caring, leisure and other service occupations	27	8·9	24	9·8	1	2·2	2	13·3
Sales and customer service occupations	9	3	7	2·9	1	2·2	1	6·7
Elementary occupations (e.g. washing, cleaning…)	1	0·3	0		1	2·2	0	
Student	24	7·9	18	7·4	6	13·3	0	
Other	19	6·3	12	4·9	7	15·6	0	
Living situation								
Alone	39	13·2	27	11·4	8	18·2	4	26·7
Wife/husband or partner	51	17·2	39	16·5	12	25	1	6·7
Wife/husband (or partner) and children	82	27·7	72	30·4	6	13·6	4	26·7
Parents	85	28·7	66	27·8	15	24·1	4	26·7
Friends	15	5·1	12	5·1	1	2·3	2	13·3
With children	16	5·4	13	5·5	3	6·8	0	
Other	8	2·7	8	3·4			0	
UK-born								
Yes	124	41·3	98	40·7	20	45·5	6	40
No	176	58·7	143	59·3	24	54·5	9	60
Co-morbidities								
Type 1 diabetes	5	3·8	4	4·0	1	3·7	0	
Type 2 diabetes	20	13·3	15	12·9	4	13·8	1	6·7
High blood pressure	8	5·4	3	2·7	4	13·8	1	6·7
Heart disease	3	2·2	1	1	2	7·1	0	
Cancer	3	2·3	2	2	1	3·8	0	

* Mixed race includes individuals of mixed Pakistani/Bangladeshi heritage as well as those of Pakistani or Bangladeshi background combined with other ethnicities.

Percentages reflect valid responses per item (i.e. excluding missing values); denominators vary by item due to differing response rates.

GCSE, General Certificate of Secondary Education.

Participants were asked whether they knew anyone with T2D. The majority (77 %) reported having close family members with the condition, 56 % mentioned extended family members and 29·4 % indicated they have friends with T2D.

Regarding medication use, 31·7 % of participants reported taking prescribed medications, primarily metformin, while 64·1 % stated they were not taking any medication. When asked to rate their overall health, 6 % described it as excellent, 27 % as very good and 56·7 % as good; 10·3 % rated their health as poor.

### Knowledge of type 2 diabetes

**Figure 1.**
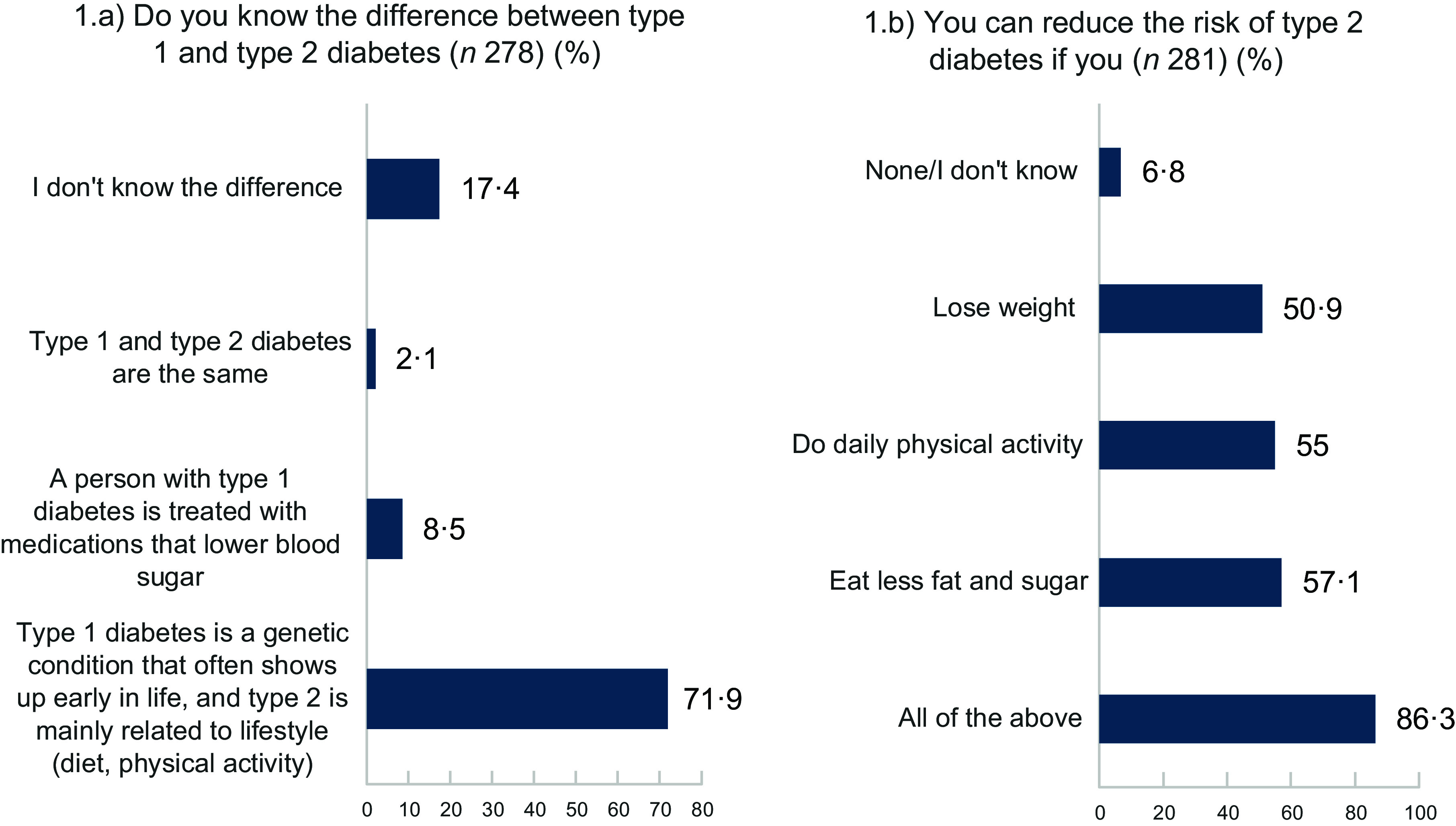
Participants’ response to their knowledge of type 2 diabetes.

The majority of participants (85·1 %) correctly identified what the condition entails. However, 14·9 % were either incorrect or unsure. Additionally, 71·9 % reported knowing the difference between type 1 and type 2 diabetes, while 17 % did not (Figure 1(a)).

Participants were asked to select all applicable options in response to the question: ‘How can you reduce the risk of developing type 2 diabetes?’; 86·3 % stated that losing weight, diet and physical activity can do (Figure 1(b)).

### Sources of information and advice regarding type 2 diabetes

Participants were asked whether they follow dietary advice provided by healthcare professionals such as doctors, nurses or dietitians. Just under a third (31·2 %) reported that they do. Only 18·8 % had consulted a nutritionist or dietitian regarding their diet. Among those who did, the primary reasons were to lose weight and manage blood sugar levels (52·8 %), while 26·4 % sought advice specifically for weight loss. When asked where they seek information about their diet, 72·1 % of participants reported using online sources such as social media and blogs. This was followed by healthcare professionals and traditional media sources (see online supplementary material, Supplemental Material 1).

### Perceived knowledge of carbohydrates

**Figure 2.**
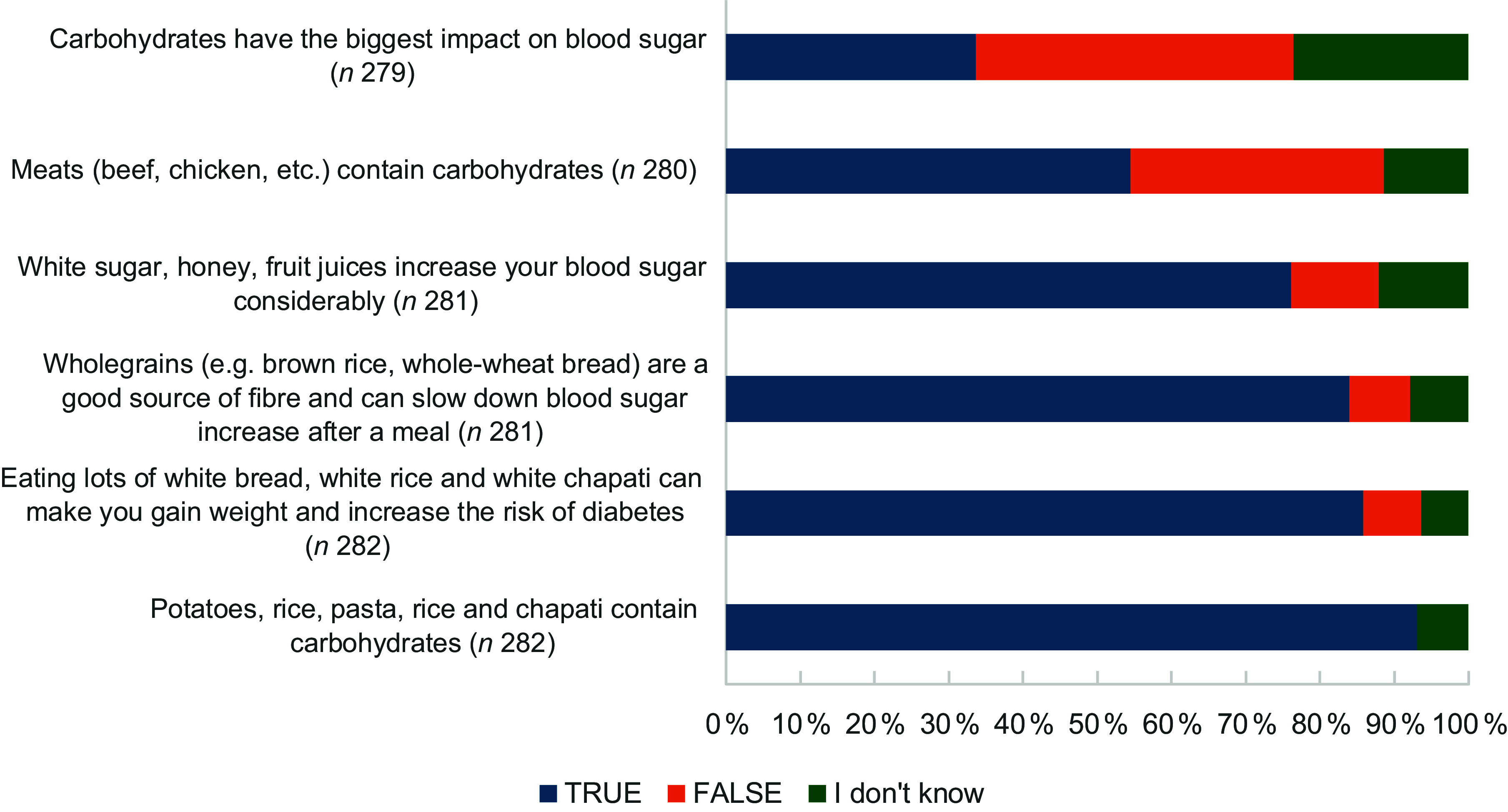
Perceived knowledge of carbohydrates.

Participants were asked about their understanding of carbohydrates; 67·7 % of participants stated they knew what the term means, while 24·5 % said they did not, and another 7·8 % reported having only a limited understanding. Although most participants demonstrated knowledge of carbohydrate sources, there were noticeable gaps in understanding the impact of carbohydrates on blood sugar levels (Figure 2).

### Key factors for following a new diet

**Figure 3.**
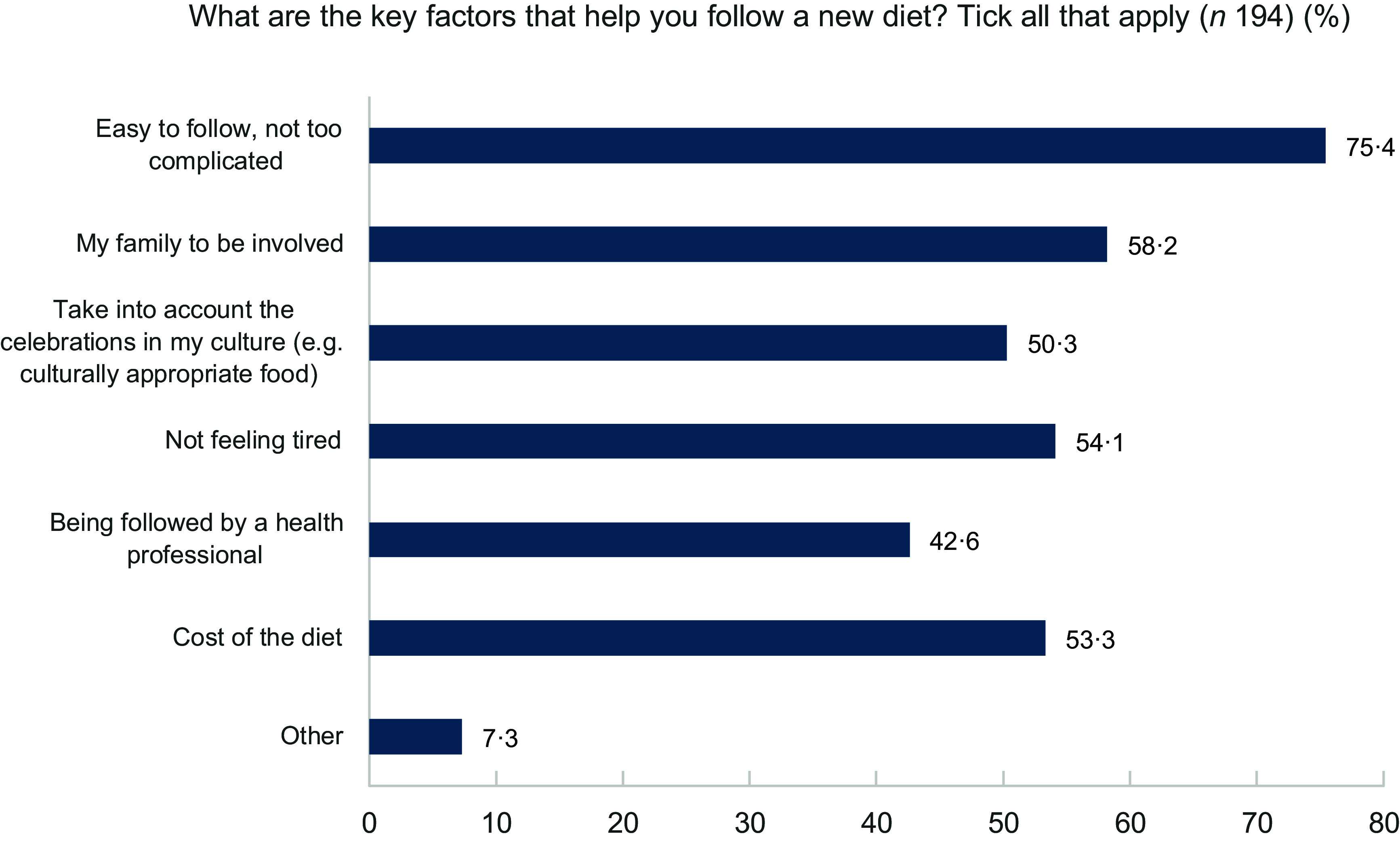
Participants’ key factors for following a new diet.

Participants were asked about key factors that help them follow a new diet. An easy-to-follow diet was the main factor (75·4 %), followed by family involvement (58·2 %), which included shared decision-making about meals, support with meal preparation and in some cases, following the same dietary plan together. Full results are added in Figure 3.

### Perceptions and knowledge regarding low-carbohydrate diets

Participants were first asked whether they had heard of or followed an LCD. Overall, 43·3 % reported having followed such a diet in the past. The decision to adopt LCD was influenced by various recommendations: advice by a health professional to lose weight (22·7 %) or manage diabetes (8·1 %), advice from family or friends to lose weight (29·8 %), manage diabetes (6·7 %) or both lose weight and prevent diabetes (12·1 %). Participants’ current and previous engagement with LCD is summarised in online supplementary material, Supplemental Material 2.

#### Opinions on low-carbohydrate diet

Participants who had previously followed an LCD were invited to share their opinions by selecting all responses that applied. Overall, 31·1 % felt the diet was either impractical or too difficult to follow, while 36·1 % stated that this diet helped them feel healthier and more confident (see online supplementary material, Supplemental Material 3).

#### Typical amount of carbohydrates consumed per meal

**Figure 4.**
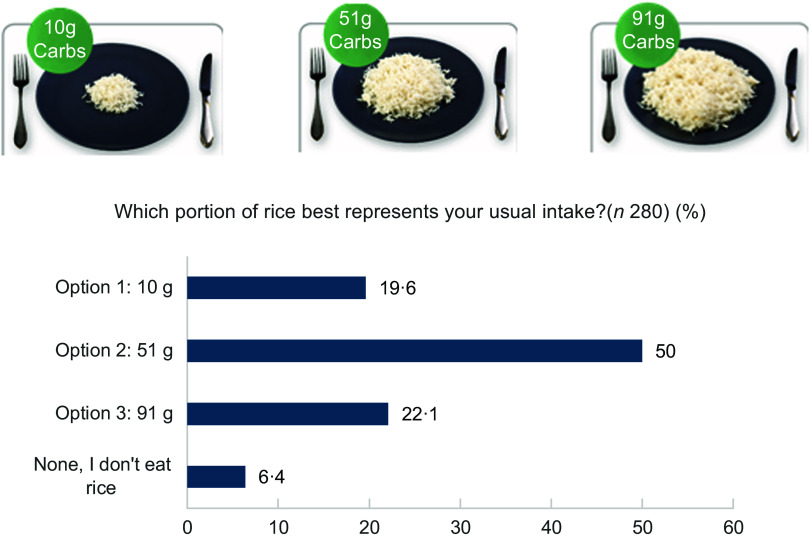
Portion of rice that corresponds to the usual intake of participants. *Source of pictures*: www.knowdiabetes.org.uk.

**Figure 5.**
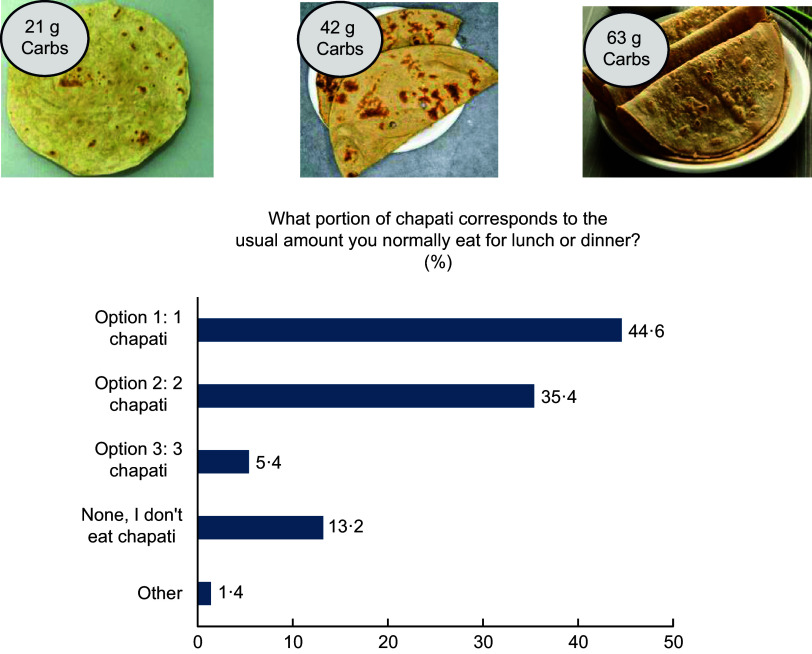
Portion of chapati that corresponds to the usual intake by participants.

To identify the typical amounts of carbohydrates consumed by participants in a meal, we used visual portion sizes of chapati and rice, two of the most commonly eaten carbohydrate sources among South Asians. Nearly a quarter (22·1 %) consumes the portion of rice that corresponds to 91 g of carbohydrate per meal, while the majority typically have 1 chapati (21 g). Results are presented in Figures 4 and 5.

#### Opinions on a typical day of 130 g of carbohydrates

Participants were shown an example of a typical daily diet containing 130 g of carbohydrates and asked whether they found it acceptable. Responses were mixed: 67·2 % reported being constrained by family meal patterns, while 34·9 % felt the portion sizes were too small (see online supplementary material, Supplemental Material 4).

Among those who selected ‘Other’, several participants provided open-ended responses offering further insight into their views. A selection of these comments is presented below:

‘Doesn’t look that nice’.

‘I have a very active job I don’t think I’d be satisfied with them portions’.

‘Time and money constraints’.

‘It’s coming out of comfort zone and have been eating from childhood and finds it hard to come out of it’.

‘Slightly difficult to follow in terms of will power. However I’m gradually trying to come towards a low carb diet’.

‘Too much stress to deal with it now’.

‘I’m too old’.

‘Too difficult to adopt as a routine’.

‘I love food and especially rice’.

### Perceptions and knowledge regarding intermittent fasting diets

Participants were first asked a series of questions exploring their opinions and perceptions of IF diets. Full results are provided in online supplementary material, Supplemental Material 5. Nearly half reported having tried IF, typically for a short duration. The 16:8 method was the most practiced, and many participants considered Ramadan fasting as a form of IF. Overall, IF was well accepted, with 76·8 % indicating they would be willing to follow it. However, when participants were asked about reasons that might prevent them from following the diet, family arrangements emerged as a notable barrier (42·2 % of participants). In particular, the expectation to take part in shared family meals was frequently cited as a challenge that made adopting IF difficult. Quotes from participants illustrating challenges in following IF are provided below.

‘Cravings’.

‘Doesn’t align with my activity levels throughout the day’.

‘Due to doctors’ advice because of heart condition’.

‘Due to type 2 diabetes. I have to have food on regular intervals and also manage that the glucose level does not exceed. So intermittent fasting is tough’.

‘From previous experience the times I prevent myself from eating for a prolonged period of time, I end up binge eating afterwards so the diet that suits me better is to have regular meals when I am hungry’.

‘Honestly it’s laziness and comfort in eating foods that I shouldn’t. The main obstacle for me is tea with a snack e.g. biscuit or pastry, etc. At work it has become a habit to have tea, I rarely eat a meal or “proper” food rather I will have a tea and/or coffee’.

‘I am a binge eater’.

‘I don’t believe in this diet’.

‘I feel again it’s changing lifestyle and coming out of comfort zone and I always need outside motivation to do something for myself and don’t have self-control’.

‘I really don’t have enough time. It’s important, but I am also lazy’.

‘I’m forgetful and can be lazy, I think meal prepping and having recipes I can follow would really help’.

‘I’m not ready yet’.

‘I’m too old now’.

‘It was only for Ramzan’.

‘Long working hours make it difficult to follow especially in summer’.

‘Medication’.

‘My mind’.

‘Never done one. So don’t know’.

‘Ramadan is only for a month in a year’.

‘While working’.

‘Work’.

Participants were asked about their preferred type of IF. The largest proportion (48·3 %) preferred time-restricted eating, such as consuming meals between 10.00 and 18.00. A smaller proportion (6·4 %) favoured the 5:2 diet, while 30·7 % expressed no specific preference. Additionally, 14·5 % stated that they would not follow any form of IF.

### Participant preferences: low-carbohydrate v. intermittent fasting diets

**Figure 6.**
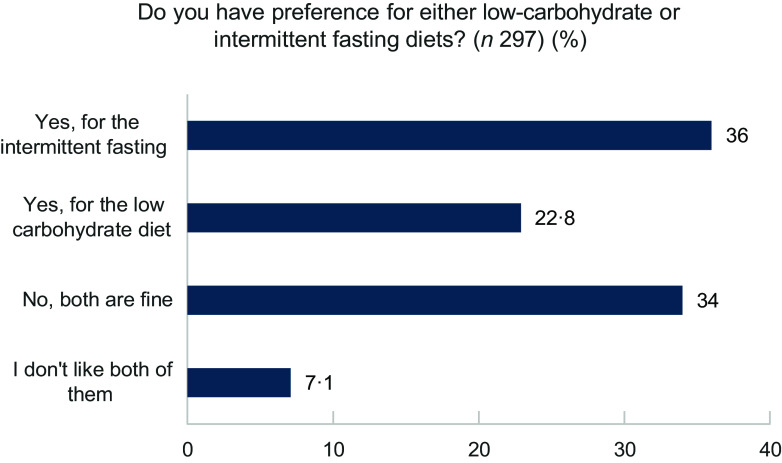
Participants’ preference for either low-carbohydrate or intermittent fasting diets.

Participants were asked whether they had a preference after receiving information on either of the specific types of diet. Responses show that answers are spread out, with IF being more acceptable than LCD (36 % *v*. 22·8 % respectively) (Figure 6).

### Opinions and feedback from the study population: qualitative data

We asked participants at the end of the survey to provide any opinions and feedback that they think could help future diet plans for people from the Pakistani/Bangladeshi background. Relevant themes and quotes are added below:

#### Cultural sensitivity and familiarity

‘Food that stays close to our culture but is also healthy. I feel like alternatives to rice and chapati need to be promoted’.

‘Incorporate a diet plan which consists of food from our households, i.e. chapatti – curry – rice etc.’.

‘It should involve around our culture, like we like to eat curries and rice a lot. So it should be included, but with the possibility of less portion size or healthier alternative options’.

#### Low-carb and intermittent fasting

‘A combination of Keto diet/low carb diet and intermittent fasting will reduce the risk of diabetes, losing weight and keep energetic’.

‘I think the ketogenic diet is perfect combined with intermittent fasting you can have curry everyday on this diet and that’s why Asian people struggle to lose weight served with keto chapati’.

‘Intermittent fasting is something that can be well adopted by people with Pakistani/Bangladeshi background or anyone who has followed the Islamic month of Ramadan fasting. I think building on this to make some alterations that can perhaps make it easier’.

#### Portion control

*‘*We love our food. The most we can do is reduce its amount’.

‘I think people need to be made aware of the actual amount of carbs in chapati and rice and understand what happens when glucose enters the bloodstream. Pakistani culture has a big focus on entertaining guests and there is an emphasis on serving guests fried foods. We need to educate ourselves on actual food portions since we have a tendency to serve in big trays and to help ourselves not realising how much excess amount we have put in for ourselves’.

#### Education

‘Education around reducing rice consumption is a necessity. Bangladeshi people are not aware of the risks associated with this. Also eating late at night is a common problem. Bangladeshis generally eat dinner around 9–10 pm. Also, more exercise and walking need to be encouraged’.

‘Important to give advice on recipes and meals to cook’.

#### Economic and practical barriers

‘Consider income of individuals. Healthier foods are often more expensive and less affordable’.

#### Family-centred eating

‘Bangladeshi meals are family orientated so needs to be taken into consideration. How to manage individual diet around family is difficult’.

‘The main issue is having a healthy meal when parents make curries. How much roti has an effect in weight/carbs and glucose spikes’.

#### Behaviour change and motivation

‘Consisting to a particular diet is very difficult. No intervention can help unless that person is self-motivated’.

‘Definitely emphasising alternatives to white rice/basmati/chapati, at present most of the older people don’t want to eat anything that isn’t rice or chapati’.

#### Gender roles in cooking

‘More education for the womenfolk and family who decide the family meal’.

#### Traditional knowledge v. western nutrition

‘We are skeptical of western understandings of nutrition… for example, the use of Ghee in cooking’.

‘Knowledge of low carbs’.

#### Other

‘None, its subjective, works differently for each person’.

### Impact of age, education and other factors on dietary knowledge and preferences

A multinomial logistic regression was conducted to assess whether predictor variables (age, gender, place of birth and education) affect participants’ knowledge. Two models were statistically significant. First, for the question ‘knowledge of the difference between type 1 and T2D’, education was a significant predictor: individuals without formal qualifications were significantly less likely to answer this question correctly (B = −4·01, *P* = 0·003, 95 % CI (0·001, 0·26)), while gender, place of birth and age were NS predictors. Similarly, when asked whether they understood the term ‘carbohydrate’, education showed a significant effect, with those having no formal qualifications less likely to answer Yes (B = −3·8, *P* = 0·01, 95 % CI (0·001, 0·449)).

We looked at preferences towards diets while considering predictors (age, gender, education, place of birth and living arrangement). Age and living arrangements were significant predictors of preference for an LCD. Younger individuals were more likely to choose this option, specifically those aged 25–34 years (B = 2·43, *P* = 0·03, 95 % CI (1·34, 96·44)) and 35–44 years (B = 2·31, *P* = 0·03, 95 % CI (1·24, 81·73)). Additionally, living with parents or with family and children was a significant predictor of the choice of this type of diet.

Additionally, participants aged 25–54 years and those living with parents were significantly more likely to express a preference for IF diets (*P* < 0·05). Similarly, individuals in this age group who lived with a partner and children or with parents and children were more likely to find both low-carbohydrate and IF diets acceptable (*P* < 0·05).

## Discussion

This study aimed to explore perceptions, knowledge and preferences regarding LCD and IF dietary interventions among Pakistani and Bangladeshi adults residing in the UK. It aimed to provide insights into how social and demographic factors influence dietary behaviours and openness to specific nutritional strategies within these communities.

While participants demonstrated a good general understanding of carbohydrates and their relevance to T2D, notable gaps remain regarding the impact of carbohydrate intake on blood glucose regulation and portion control. With educational programmes having consistently been associated with improved diabetes outcomes^(23,24)^, future efforts must prioritise guiding individuals towards accessible, evidence-based resources, particularly given that 72·1 % of participants reported relying primarily on social media and blogs for information, which often lack credibility. However, education alone may not be sufficient. Qualitative studies involving British South Asian populations have revealed that, despite possessing adequate knowledge, many individuals remain resistant to behavioural change^(18)^, highlighting the need to look beyond education to address motivational, social and cultural barriers.

A notable finding was the relatively high level of acceptability of IF diets, with 76·8 % of participants expressing willingness to follow such an approach and 36 % preferring it over LCD. This reinforces the notion that cultural familiarity with fasting may have contributed to the greater acceptability of IF interventions^(22)^. While LCD were less acceptable, they still attracted interest, particularly among younger participants (25–34 years). Nevertheless, the prescribed intake of 130 g of carbohydrate elicited mixed responses, suggesting the importance of considering portion size limitations and family meal dynamics when designing such dietary plans.

Shared family meals and household routines can pose significant barriers to dietary adherence among Pakistani and Bangladeshi populations, as evidenced in this study and supported by previous research^(25,26)^. Communal meal timing and content, coupled with resistance to altering established dietary habits and limited motivation, highlight the need for culturally sensitive strategies. Framing dietary interventions within familiar cultural contexts may therefore enhance their acceptability and effectiveness. Additionally, current government health guidelines lack cultural responsiveness^(27)^. They focus on individualised dietary advice, overlooking the communal nature of eating in South Asian households, where meal decisions are often made collectively. This gap reduces the effectiveness of interventions in these communities. To address this, strategies should actively involve family members in nutritional education, encourage healthy food choices and portion control and provide flexible options for meal timing. In particular, the role of meal providers, often women, should be recognised as they exert significant influence over household food choices and preparation^(28)^. Interventions that empower and educate these key decision-makers may have a greater impact on adherence. Without integrating these considerations into educational strategies and intervention design, even well-structured dietary plans may face challenges in achieving sustained adherence.

Another critical issue is the limited understanding of South Asian dietary patterns among UK health professionals, which can lead to generic advice that overlooks cultural norms, traditional foods and communal eating practices. Previous studies have highlighted this lack of cultural competence, emphasising the need for professional training and resources that are tailored to the needs of diverse populations^(29,30)^.

With this study largely reinforcing existing knowledge, what is now needed is a clear, actionable plan to translate insights into effective practice. Our findings support the rationale for randomised controlled trial to evaluate the effects of IF interventions on glycaemic control, first in terms of T2D prevention but also management, IF being a safe and effective strategy for improving glycaemic outcomes and supporting weight loss in individuals with T2D^(20,21)^. A multidisciplinary approach involving healthcare professionals, educators and community leaders will be essential to developing sustainable, inclusive strategies that promote long-term adherence and improved outcomes. Greater attention should be directed towards older adults (over 54 years) to explore what adaptations might improve acceptability within that demographic.

This study has several limitations that should be acknowledged. First, not all survey questions were compulsory, which led to some missing responses. Second, the sample predominantly involved educated and young individuals, which may limit the generalisability of the findings to the wider Pakistani and Bangladeshi populations in the UK, particularly those with lower levels of formal education. Third, we made every effort to reduce language barriers by including both English and non-English speakers, yet participation from non-English speakers remained limited. Furthermore, due to the relatively small number of Bangladeshi participants, it was not possible to conduct meaningful comparisons between subethnic groups. Dietary habits between the Pakistani and Bangladeshi populations are known to slightly differ, and as a result, potential differences in dietary perceptions and practices between the two communities could not be explored in depth. Nonetheless, this limitation reflects demographic realities, as the UK Bangladeshi population is smaller than its Pakistani counterpart. Lastly, although we asked about living arrangements, we did not specifically consider multigenerational households, which may have provided greater clarity and deeper insight into dietary influences and decision-making dynamics. Future studies should aim to recruit a more diverse and representative sample, ensure consistent response rates across survey items and explore subethnic variations to better understand the distinctions within South Asian populations.

Overall, IF appeared to be more favourably received among a sample of Pakistani and Bangladeshi people. This supports the rationale for designing future trials targeting this population, with IF interventions that are culturally relevant and practically flexible. Future research should also aim to explore subethnic differences, include participants from a broader range of educational backgrounds and assess the long-term feasibility and sustainability of these dietary strategies.

## Supporting information

10.1017/S136898002610250X.sm001 Farhat et al. supplementary material 1 Farhat et al. supplementary material

10.1017/S136898002610250X.sm002 Farhat et al. supplementary material 2 Farhat et al. supplementary material

10.1017/S136898002610250X.sm003 Farhat et al. supplementary material 3 Farhat et al. supplementary material

10.1017/S136898002610250X.sm004 Farhat et al. supplementary material 4 Farhat et al. supplementary material

10.1017/S136898002610250X.sm005 Farhat et al. supplementary material 5 Farhat et al. supplementary material

## Supplementary material

For supplementary material accompanying this paper, visit https://doi.org/10.1017/S136898002610250X.
