# Supplementary material for: Dietary knowledge and perceptions in UK Pakistani/Bangladeshi communities: a survey on low-carbohydrate and intermittent fasting diets for diabetes prevention and management
Source: Public Health Nutr. 2026 Apr 10;29(1):e103. doi: 10.1017/S136898002610250X (PMC13434497; doi:10.1017/S136898002610250X)
Supplement: Supplementary file 1 [file S136898002610250Xsup001.docx]

Where do get your information/advice about your diet? Tick all that apply (n=304) (%)

|  | % |
| --- | --- |
| Other | 6 |
| Media (TV, radio, newspapers/magazines) | 31.8 |
| Family | 40.5 |
| No where | 41 |
| Healthcare professionals | 53.6 |
| Online (socia media, blogs) | 72.1 |

**Supplementary material 1: Sources of dietary information as reported by participants**
