# Supplementary material for: Dietary knowledge and perceptions in UK Pakistani/Bangladeshi communities: a survey on low-carbohydrate and intermittent fasting diets for diabetes prevention and management
Source: Public Health Nutr. 2026 Apr 10;29(1):e103. doi: 10.1017/S136898002610250X (PMC13434497; doi:10.1017/S136898002610250X)
Supplement: Supplementary file 2 [file S136898002610250Xsup002.docx]

**Supplementary material 2: Participants’ practices of low-carbohydrate diets**

|  | **Yes** | **No** |
| --- | --- | --- |
| Have you heard of Atkins diet? (n=281) | 21% | 79% |
| Zone diet (n=157) | 21% | 79% |
| Keto diet (n=174) | 65.9% | 34.1% |
| Low carbohydrate diet (130g) (n=227) | 58.5% | 41.5% |
| **Have you followed:** | **Followed it in the past** | **Following it now** |
|  |  |  |
| A low carbohydrate diet? (130g)  (n=282) | 43.3% | 5% |
| Atkins diet (n=180) | 5% | 0.8% |
| Zone diet (n=190) | 14.9% | 0.8% |
| Keto diet (n=121) | 14.9% | 2.7% |
