# Supplementary material for: Dietary knowledge and perceptions in UK Pakistani/Bangladeshi communities: a survey on low-carbohydrate and intermittent fasting diets for diabetes prevention and management
Source: Public Health Nutr. 2026 Apr 10;29(1):e103. doi: 10.1017/S136898002610250X (PMC13434497; doi:10.1017/S136898002610250X)
Supplement: Supplementary file 3 [file S136898002610250Xsup003.docx]

What is your opinion on low-carbohydrate diets? (n=119) (%)

|  | % |
| --- | --- |
| Too difficult | 13.4 |
| Not practical for myself and family | 17.7 |
| It was acceptable | 32.8 |
| I felt healthier and more confident | 36.1 |

**Supplementary material 3: Participant’s opinions on following a low-carbohydrate diet**
