# Supplementary material for: Dietary knowledge and perceptions in UK Pakistani/Bangladeshi communities: a survey on low-carbohydrate and intermittent fasting diets for diabetes prevention and management
Source: Public Health Nutr. 2026 Apr 10;29(1):e103. doi: 10.1017/S136898002610250X (PMC13434497; doi:10.1017/S136898002610250X)
Supplement: Supplementary file 4 [file S136898002610250Xsup004.docx]

What issues or barriers would stop you from following this diet? Tick all that apply (%)

|  | % |
| --- | --- |
| Other | 17.4 |
| No barriers, I would be interested in following it | 59.3 |
| Too little amounts | 34.9 |
| I eat whatever meals my family prepares | 67.2 |

**Supplementary material 4: Opinions and barriers on the adoption of a low CARBOHYDRATE diet (130g)**
