# Supplementary material for: Dietary knowledge and perceptions in UK Pakistani/Bangladeshi communities: a survey on low-carbohydrate and intermittent fasting diets for diabetes prevention and management
Source: Public Health Nutr. 2026 Apr 10;29(1):e103. doi: 10.1017/S136898002610250X (PMC13434497; doi:10.1017/S136898002610250X)
Supplement: Supplementary file 5 [file S136898002610250Xsup005.docx]

**Supplementary material 5: Knowledge and perceptions regarding intermittent fasting diets**

**Table S5.1. Awareness of intermittent fasting diet**

| Question | Yes (%) | No (%) |
| --- | --- | --- |
| Have you heard of intermittent fasting before? | 88.3 | 11.7 |

**Table S5.2. Previous use of intermittent fasting**

| Question | Yes (%) | No (%) |
| --- | --- | --- |
| Have you followed any type of intermittent fasting before? | 49 | 51 |

**Figure S5.1. Types of intermittent fasting diets followed by participants**

**Figure S5.2. Duration of intermittent fasting practice followed by participants**

**Table S5.3. Perceived ability to follow intermittent fasting (n=207).**

| Question | Yes, if that helps prevent or manage diabetes (%) | No (%) |
| --- | --- | --- |
| If you haven’t followed one yet, based on the definitions of the different types of intermittent diet listed previously, do you feel this is a diet you might be able to follow? | 76.8 | 23.2 |

What reasons would stop you from following an intermittent fasting diet? (Tick all that apply) (n=211)(%)

|  | % |
| --- | --- |
| Other | 18.5 |
| Too difficult | 43.2 |
| I am committed to my family's meal time and meal patterns | 42.2 |
| None, I would be happy to do it | 71.6 |

**Figure S5.3. Reasons that would stop participants from following an intermittent fasting diet**

*Note: Percentages may not sum to 100% where participants could select more than one response (if applicable) or due to rounding.*
